# Prevalence, MRI findings, and clinical features of lumbosacral intervertebral disc protrusion in French Bulldogs diagnosed with acute thoracic or lumbar intervertebral disc extrusion

**DOI:** 10.3389/fvets.2023.1302418

**Published:** 2023-11-23

**Authors:** Claudia La Rosa, Simona Morabito, Andrea Carloni, Tommaso Davini, Carlotta Remelli, Swan Specchi, Marco Bernardini

**Affiliations:** ^1^AniCura I Portoni Rossi Veterinary Hospital, Bologna, Italy; ^2^Antech Imaging Services, Fountain Valley, CA, United States; ^3^Department of Animal Medicine, Productions and Health, University of Padua, Legnaro, Italy

**Keywords:** congenital vertebral malformations, chondrodystrophic dogs, intervertebral disc protrusion, intervertebral disc extrusion, lumbosacral junction, magnetic resonance imaging

## Abstract

**Introduction:**

Intervertebral disc protrusion (IVDP) is a neurological disorder commonly observed at the lumbosacral junction of old, medium-to-large breeds, non-chondrodystrophic dogs. Although uncommon, lumbosacral IVDP can also be seen in chondrodystrophic dogs, among them French Bulldogs (FBs) and could be associated with congenital vertebral malformations in this breed. This study aims to evaluate the prevalence, clinical features, and MRI characteristics of lumbosacral IVDP and congenital vertebral malformations in FBs diagnosed with thoracic or lumbar intervertebral disc extrusion (IVDE) and to evaluate the possible interference of the neurologic deficits related to chronic IVDP on neurological examination.

**Materials and methods:**

This is a single-center, retrospective case series. A search for FBs diagnosed with IVDE affecting the thoracic or lumbar regions is done on the database of the AniCura I Portoni Rossi Veterinary Hospital (Zola Predosa, Bologna, Italy). Eligible dogs have a complete medical report and a high-field MRI of the lumbosacral junction. MRIs of the lumbosacral junction are evaluated to determine the position of IVDP, cranial intervertebral foraminal stenosis, and signs of nerve root involvement. Radiographs, when available, are reviewed to identify the presence of lumbosacral congenital vertebral malformations.

**Results:**

Eighty FBs are included in the study. The prevalence of lumbosacral IVDP among FBs is 91.3%. Among FBs with lumbosacral IVDP, 45.0% show concurrent cranial intervertebral foraminal stenosis, 28.8% exhibit concurrent nerve root involvement, 56.2% appear to be asymptomatic for lumbosacral changes, while 15.1% manifest a decreased or absent withdrawal reflex as a supposed consequence of chronic lumbosacral IVDP. Congenital vertebral malformations are detected in 10 dogs.

**Conclusion:**

The results of this study support the hypothesis that lumbosacral IVDP is frequent in FBs presenting with thoracic or lumbar IVDE. In over half the dogs lumbosacral IVDP appears to be asymptomatic; however, in other cases, chronic lumbosacral IVDP seems to cause neurological deficits that may lead to erroneous localization of acute IVDE, representing a confounding factor for clinicians.

## 1 Introduction

Intervertebral disc herniation (IVDH) is a well-documented pathological condition in dogs, encompassing intervertebral disc extrusion (Hansen type I herniation; IVDE) and intervertebral disc protrusion (Hansen type II herniation; IVDP) (1–5). IVDE is most commonly seen in chondrodystrophic (CD) breeds affecting the cervical and thoracolumbar regions (3, 5). French Bulldogs (FBs) are highly prone to developing IVDE, with a reported prevalence of 45.5% among all neurological conditions (6).

In contrast, IVDPs are commonly observed in older, medium-to-large, non-chondrodystrophic (NCD) breeds and broadly represent the most common type of IVDH affecting the lumbosacral (LS) junction (2, 4, 7–10). LS IVDP can result in compressive radiculopathy of the cauda equina and intervertebral foraminal stenosis. Abnormal findings upon neurological examination are LS pain, paresthesia, proprioceptive deficits, lameness of the pelvic limbs, stiffness during physical activity, difficulty in jumping, a depressed withdrawal reflex and muscle atrophy, especially of the gluteal and hamstring muscle groups (8, 11, 12).

Albeit uncommon, IVDP can occur in CD dogs. IVDPs affecting the LS junction are poorly reported in CD dogs (4). A recent study found 72/149 dogs with LS IVDH within a population of neurologically normal FBs, English Bulldogs, and pugs; among FBs alone, the same study recorded a prevalence of 52.8% for LS IVDH (13). Another study reported a prevalence of 77.4% within a population of FBs, both with and without neurological disorders (14).

Furthermore, the LS junction has been reported to be affected by congenital vertebral malformations (CVMs) in CD dogs based on CT examination and a possible association between CVMs and LS IVDP has been suggested (13, 14).

To the best of our knowledge, no studies have focused on MRI characteristics of LS IVDP and the resulting involvement of nerve roots in FBs. It is the authors’ opinion that LS IVDP in FBs is a frequent, underdiagnosed, chronic condition that can cause neurological deficits possibly interfering with neurological evaluation when these dogs are presenting with acute thoracic or lumbar IVDE. Therefore, this study aims to evaluate (1) the prevalence and MRI characteristics of LS IVDP, cranial intervertebral foraminal stenosis, root involvement, and CVMs in FBs diagnosed with thoracic or lumbar IVDE and (2) the possible interference of the neurologic deficits related to chronic IVDP on neurological examination performed when dogs are evaluated for acute IVDE.

It is hypothesized that a high prevalence of LS IVDP may be a confounding factor at the time of neuroanatomical localization of acute myelopathy and an association between LS CVM and IVDP may exist.

## 2 Materials and methods

### 2.1 Case recruitment criteria

This is a single-center, descriptive, retrospective case series. The database of the AniCura I Portoni Rossi Veterinary Hospital (Zola Predosa, Bologna, Italy) was searched for FBs that were diagnosed through MRI with IVDE affecting the thoracolumbar (T3–L3) or LS (L4–S3) regions between April 2018 and February 2023. All the animals used in the study were client-owned and underwent MRI examinations of the vertebral column as part of their diagnostic workup. Dogs were eligible if they had (1) a medical report including signalment and a neurological examination performed by a European College of Veterinary Neurology (ECVN) board-certified veterinary neurologist or an ECVN resident and (2) an MRI of the vertebral column extended to the LS junction, imaged in the T2-weighted sagittal plane and at least one other plane. If multiple MRI studies of the same dog were performed at different times, only the oldest one including the LS junction was examined. Patients with multiple IVDEs, LS IVDE, or other concurrent pathological conditions affecting the spinal cord or acquired disease processes of the vertebral column between T3 and S3 were excluded. Radiographs of the LS junction were also evaluated when available.

### 2.2 Medical records review

Details regarding signalment (sex, age, and neutered/spayed status), neurological examination, and location of IVDE were collected. Information obtained from neurological examination included: (1) analysis of the gait graded from 1 (spinal hyperesthesia only) to 5 (paraplegia without deep pain) according to a grading system described elsewhere (15); (2) assessment of the withdrawal reflex of pelvic limbs (normal/increased or reduced/absent); (3) level of cutaneous trunci muscle reflex (CTMR) cut-off; and (4) assessment of thoracic/lumbar or LS pain (present or absent). Dogs were divided into two groups based on the localization of IVDE: group A (cranial to the fourth lumbar vertebra [L4]), and group B (caudal to L4). Dogs in both groups were further subdivided into cases with normal withdrawal reflexes and cases with reduced/absent withdrawal reflexes.

### 2.3 Images analyses

Radiographs and MRI were reviewed by two European College of Veterinary Diagnostic Imaging board-certified radiologists (A.C. and S.M.), two interns (C.L.R. and T.D.), and an ECVN board-certified neurologist (M.B.) using a DICOM viewer program (Horos DICOM viewerTM).^1^ All the reviewers were blinded about the findings of neurological examinations.

**Figure 1:**
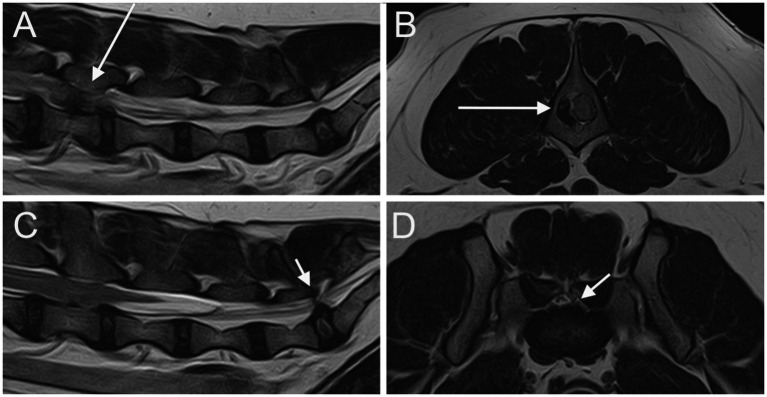
Parasagittal **(A)** and sagittal **(C)** T2W images of the lumbar vertebral column and the LS junction; transverse T2W at the level of L4 vertebra **(B)** and at the level of LS junction **(D)**. A severe extrusion of the L4-L5 IVD in seen on the right side of the vertebral canal (long arrow). An IVDP affecting both the vertebral canal and the intervertebral foramina is seen at the LS junction (short arrows).

**Figure 2:**
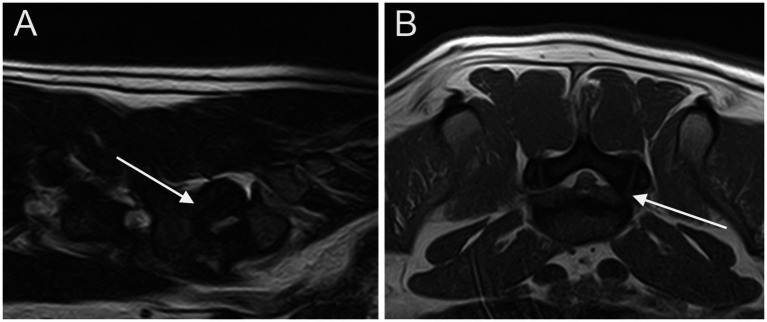
Parasagittal **(A)** and transverse **(B)** T2W images of the LS junction showing cranial intervertebral foraminal stenosis (arrows).

**Figure 3:**
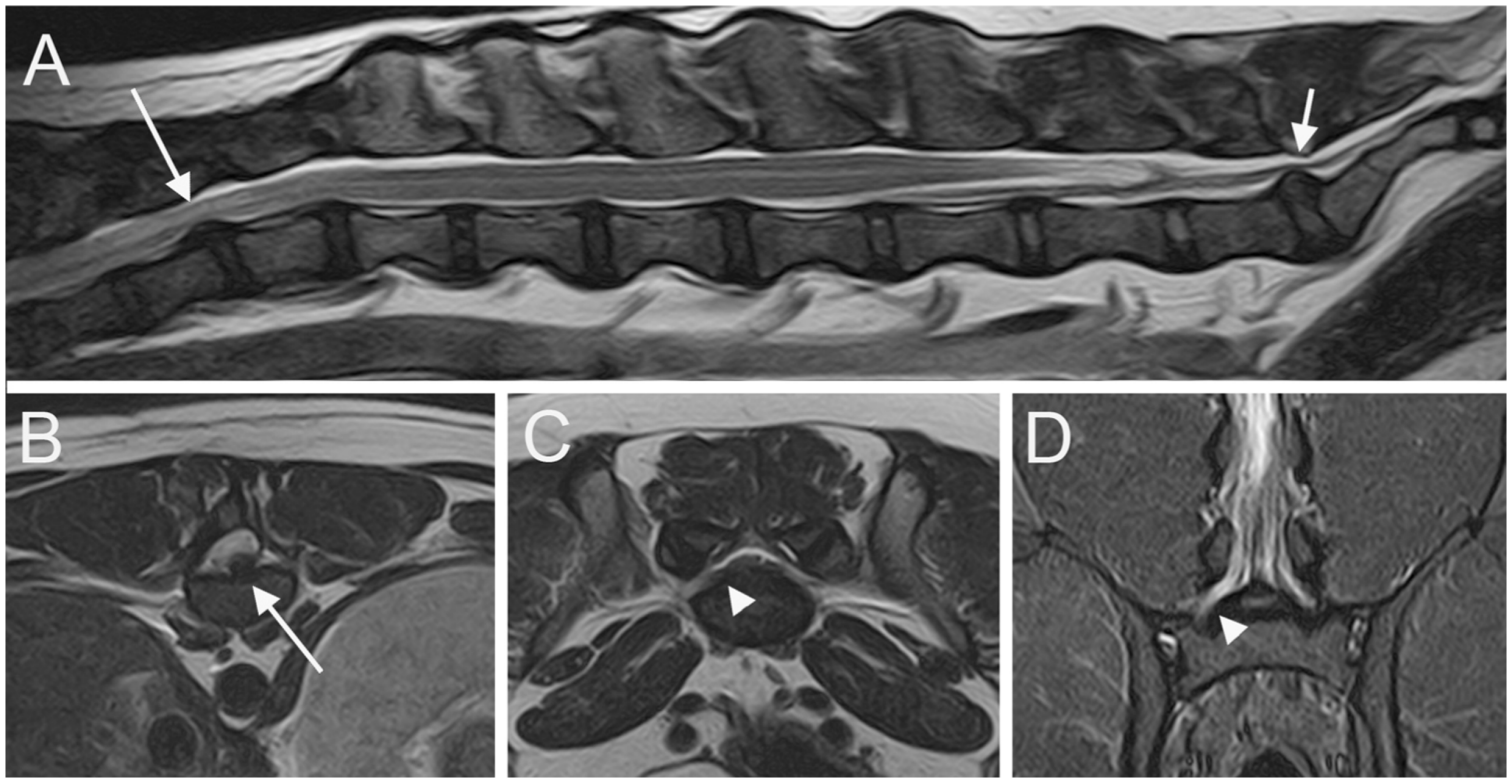
Sagittal T2W image **(A)** of the T11-S1 vertebral column; transverse T2W images at the level of L4 caudal epiphysis **(B)** and at the level of LS junction **(C)**; dorsal STIR **(D)** image of the L6-S1 vertebral column. A T12-T13 IVDE is seen (long arrows); a LS IVDP (short arrow) is associated with an enlargement and signal change of the right L7 nerve root (arrowheads).

MRI scans of the LS junction were evaluated for LS IVDP, cranial intervertebral foraminal stenosis, and involvement of spinal nerve roots. IVDP was classified as canalar, left or right intervertebral foraminal, bilateral intervertebral foraminal, canalar + right/left intervertebral foraminal, and all positions (canalar + bilateral intervertebral foraminal) (Figure 1). The degree of LS IVDP was scored as grade 1 when obstruction of the spinal canal was <25%, grade 2 when obstruction was 25–50%, and grade 3 when the obstruction was >50% (10, 16). Cranial intervertebral foraminal stenosis was defined as an abnormal conformation and/or signal changes of the edge of the cranial part of the LS intervertebral foramen leading to a reduction in size (Figure 2). Spinal nerve root involvement was defined as occurring when nerve roots were compressed, enlarged, showed MRI signal changes, or were not detected due to the presence of degenerative material along the nerve root path (Figure 3) and estimated as absent, left/right lateral, or bilateral. Radiographs were reviewed for the presence of LS CVM such as butterfly vertebra, transitional vertebra, block vertebrae, and spina bifida. When radiographs were not available, we analyzed MRI for the same alterations. The above findings were recorded in a spreadsheet (Microsoft Excel for Mac, Version 16.74). A descriptive analysis was performed by C.L.R. for all these variables: IVDPs at the level of the intervertebral foramina, cranial intervertebral foraminal stenosis, spinal nerve root involvement, and LS CVM were classified as present or absent; IVDPs at the level of spinal canal were classified as absent, grade 1, grade 2 or grade 3 as described above.

## 3 Results

### 3.1 Case recruitment criteria and medical records review

Initially, 173 dogs were considered for inclusion. Seven cases were excluded because of an incomplete medical report, 77 cases because the LS junction was either not included in the MRI or not imaged as required, and 9 cases because of concurrent pathological conditions affecting the spinal cord, multiple IVDEs, or LS IVDE. Consequently, 80 FBs fulfilled the inclusion criteria. These FBs comprised 54 males (67.5%) of which 10 were neutered and 26 females (32.5%) of which 21 were spayed. Age at presentation ranged from 15 months to 9 years, with a mean of 2.9 years and a median age of 4 years.

Neurological examination findings and sites of IVDE are summarized in Table 1. Forty-nine out of 80 dogs (61.3%) were diagnosed with an IVDE cranial to the fourth lumbar vertebra L4 (group A), and 31/80 dogs (38.8%) with an IVDE caudal to L4 (group B). Within group A, 34/49 dogs (69.4%) had a normal withdrawal reflex bilaterally and 15/49 dogs (30.6%) had bilateral (12/15; 80%) or unilateral (3/15; 20%) reduced/absent withdrawal reflex. Within group B, 12/31 dogs (38.7%) had bilaterally normal withdrawal reflexes, while 19/31 dogs (61.3%) had either bilateral (16/19; 84.2%) or unilateral (3/19, 15.7%) reduced/absent withdrawal reflex.

**Table 1:** Distribution of thoracic and lumbar IVDE and clinical neurological findings in all dogs included.

	*N* = 80 (%)
**Neurological findings**	
Gait	
Grade 0	5 (6.3%)
Grade 1	6 (7.5%)
Grade 2	42 (52.5%)
Grade 3	15 (18.8%)
Grade 4	8 (10%)
Grade 5	4 (5%)
**Spinal reflexes**	
Withdrawal reflex	
Reduced/absent	34/80 (42.5%)
Bilateral	28/34 (82.4%)
Left	4/34 (11.8%)
Right	2/34 (5.9%)
CTMR cut-off	21/80 (26.3%)
**Pain**	54/80 (67.5%)
Thoracic or lumbar	50/54 (92.6%)
Lumbosacral	4/54 (5%)
**Neurolocation (IVDE site)**	
T11–T12	1 (1.3%)
T12–T13	4 (5%)
T13–L1	4 (5%)
L1–L2	10 (12.5%)
L2–L3	12 (15%)
L3–L4	18 (22.5%)
L4–L5	19 (23.8%)
L5–L6	11 (13.8%)
L6–L7	1 (1.3%)

### 3.2 Imaging analysis

Radiographs were available for review in 70/80 cases (87.5%), with both lateral and ventrodorsal projections available in 52/70 cases (74.3%), and only 1 projection in 18/70 cases (25.7%). MRI was conducted under general anesthesia, with dogs placed in dorsal recumbency. All studies were performed with a 1.5T MR scanner (Vantage Elan, Canon Medical Systems Europe B.V., Netherlands) with the best-fitting 16-channel flexible receiving coil from the 2 available. MRI of the LS junction was available in 2 planes in 53 cases (66.3%) (48 cases on sagittal and dorsal planes and 5 cases on sagittal and transverse planes) and in all 3 spatial planes in 27 cases (33.7%). MRI sequences available for review are summarized in Supplementary Table 1.

At least one abnormality (IVDP, cranial intervertebral foraminal stenosis, or nerve root involvement) at the level of the LS junction was observed in 76/80 dogs (95%). Of the 76 dogs, 6 (7.9%) showed all the 3 types of LS abnormalities bilaterally. Seventy-three out of 76 dogs (96.1%) presented with LS IVDP in at least 1 position, and 45% of these dogs (33/73) showed concurrent cranial intervertebral foraminal stenosis while 28.8% of these dogs (21/73) had concurrent nerve root involvement. IVDPs that affect all the positions were observed in most patients (53/73; 72.6%) (Figure 1), less frequently at both the canalar and unilateral intervertebral foraminal level (11/73; 15.1%) and rarely at the intervertebral foraminal position (7/73; 9.6%) or the canalar position (2/73; 2.7%). LS IVDPs were graded 1 in 34/73 dogs (46.6%), 2 in 19/73 dogs (26.0%), and 3 in 20/73 dogs (27.4%). Moreover, 36 out of 76 cases (47.4%) showed cranial intervertebral foraminal stenosis (Figure 2), while 37 cases (48.7%) presented with the involvement of spinal nerve roots in at least 1 side (Figure 3).

In both group A and group B, IVDPs affected at least one position (43/49, 87.8% and 30/31, 96.8%, respectively). In both groups, most IVDPs affected all the positions, while IVDPs at both canalar and unilateral intervertebral foraminal level, or bilateral intervertebral foraminal level, or canalar position or unilateral intervertebral foraminal level were seen less frequently. The distribution of IVDP, cranial intervertebral foraminal stenosis, and nerve root involvement within the population and within each group is summarized in Table 2.

**Table 2:** LS changes within the entire population.

LS changes	Entire population n. of dogs (%)	Group A n. of dogs (%)	Group B n. of dogs (%)
IVDP	73 (91.3%)	43 (87.8%)	30 (96.8%)
Canalar IVDP	2 (2.7%)	2 (4.7%)	0 (0%)
Left intervertebral foraminal	2 (2.7%)	1 (2.3%)	1 (3.3%)
Right intervertebral foraminal	0 (0%)	0 (0%)	0 (0%)
Bilateral intervertebral foraminal	5 (6.8%)	3 (7%)	2 (6.7%)
Canalar + left intervertebral foraminal	5 (6.8%)	4 (9.3%)	1 (3.3%)
Canalar + right intervertebral foraminal	6 (8.2%)	2 (4.7%)	4 (13.3%)
All positions	53 (72.6%)	31 (72.1%)	22 (73.3%)
Cranial intervertebral foraminal stenosis	36 (45%)	22 (44.9%)	14 (45.2%)
Left lateral	5 (13.9%)	1 (4.5%)	4 (28.6%)
Right lateral	11 (30.6%)	5 (22.7%)	6 (42.9%)
Bilateral	20 (55.6%)	16 (72.7%)	4 (28.6%)
Nerve root involvement	37 (46.3%)	20 (40.8%)	17 (54.8%)
Left lateral	13 (35.1%)	6 (30%)	7 (41.2%)
Right lateral	7 (18.9%)	4 (20%)	3 (17.6%)
Bilateral	17 (45.9%)	10 (50%)	7 (41.2%)

CVMs were detected in 10 cases (12.5%). A butterfly vertebra affecting the last lumbar and the first sacral vertebra was seen in 3 and 4 cases, respectively. An LS transitional vertebra was observed in 3 cases. No dogs presented with spina bifida or block vertebrae. All the 10 dogs with CVM had a concurrent LS IVDP.

### 3.3 Association between clinical and MRI findings

Among the 34 dogs in group A with a bilaterally normal withdrawal reflex, 30 (88.2%) showed LS IVDP with or without concurrent LS alterations. Of the remaining 4 dogs, 2 (5.9%) revealed cranial intervertebral foraminal stenosis without spinal nerve root involvement, and the other 2 (5.9%) displayed no LS abnormalities. Among the 15 dogs in group A with decreased/absent withdrawal reflex, 13 (86.7%) manifested LS IVDP with or without concurrent LS alterations; in this subgroup, 7 dogs were neurologically graded 2, 4 dogs were neurologically graded 3, and 2 dogs were neurologically graded 4. One of the two remaining dogs exhibited only cranial intervertebral foraminal stenosis without spinal nerve root involvement and the other showed no LS abnormalities; these two dogs were neurologically graded four and five, respectively. Among the 12 dogs in group B with bilateral normal withdrawal reflex, 11 (91.6%) showed LS IVDP and 1 showed no LS abnormalities. All 19 dogs in group B with decreased/absent withdrawal reflex showed an LS IVDP with or without concurrent LS abnormalities. Details about LS changes within each group are summarized in Table 3.

**Table 3:** Prevalence of LS changes within each group.

	Group A		Group B	
	Normal withdrawal reflexes	Impaired withdrawal reflexes	Normal withdrawal reflexes	Impaired withdrawal reflexes
IVDP	11	3	0	5
Cranial intervertebral foraminal stenosis	2	1	0	0
Nerve root involvement	0	0	0	0
IVDP + cranial intervertebral foraminal stenosis	5	4	3	5
IVDP + nerve root involvement	9	1	5	6
All LS changes	5	5	3	3
No LS changes	2	1	1	0
Total	34	15	12	19

Among the 73 dogs with LS IVDP, only 4 (5.5%) were found to have LS spinal pain upon neurological examination. All the 4 dogs revealed bilaterally decreased/absent withdrawal reflex; among these dogs, 3 were in group A and 1 was in group B. Of the remaining 69 dogs, 44 (63.8%) showed hyperesthesia at the caudal thoracic or lumbar levels that matched with the IVDE site upon MRI examination, 20 (29%) revealed no spinal pain and a bilaterally normal withdrawal reflex or a CTMR cutoff that could suggest thoracolumbar localization and 5 (7.2%) exhibited no spinal pain, decreased/absent withdrawal reflexes, and no CTMR cut-off.

## 4 Discussion

The results of this study support our hypothesis that LS IVDP is frequent in FBs presenting with thoracic or lumbar IVDE. LS IVDP affecting at least 1 position (canalar, intervertebral foraminal, or both) was observed in 91.3% of the FBs in our population, a percentage much higher than that reported in 2 previous studies [77.4%; (14) and 52.8% (13)] and more than half of the FBs in our population were graded ≥2. This difference may be due to several factors. First, the FBs in our population were older (median age = 4 years) than the dogs described in the paper written by Bertram et al. (median age 16 months) (13). Second, the positioning of the patient for MRI may have played a role. Our patients were placed in dorsal recumbency while in the study of Bertram et al. patients were examined under sternal recumbency (13). The supine position has the effect of extending the LS junction, leading to an exacerbation of IVDP with respect to the neutral position (1, 16, 17). Thus, positioning may have partially led to an overestimation of the real severity of LS IVDP in our population. However, in our study, 45% of the FBs with LS IVDP showed concomitant cranial intervertebral foraminal stenosis, a degenerative condition frequently reported with LS IVDP, and 28.8% revealed concurrent nerve root involvement. These findings decrease the likelihood of positioning-related bias (17, 18). Third, the contrast between epidural fat and a degenerated disc is stronger in MRI than in CT, allowing MRI to offer a precise detection of mild IVDPs. This property of the MRI may also be useful in defining the spatial distribution of IVDPs (3, 19). The latter point may explain the greater prevalence (87.6%) of large LS IVDPs affecting both the vertebral canal and at least one foramen in our population compared to a previous study (14).

The presence of LS CVMs has been considered to be a predisposing factor for LS IVDH. An association between LS IVDH with both LS transitional vertebra in a population of non-CD dogs (20) and hemivertebra at L7 or S1 (13) has been described. The prevalence of LS CVMs in our population study was much lower (12.5%) than that reported in recent papers based on CT examination (49.1 and 56.3% in neurological and non-neurological FBs, respectively) (13, 14) and similar to that reported in studies based on radiographs (0 to 17.5%) (21–23). We can suppose that LS IVDPs in FBs are a common finding even in absence of LS CVMs. However, MRI and radiography are not the best imaging modalities for diagnosing CVMs. CT is reported to be far more sensitive in the identification of the number of CVMs when compared to both radiography (24, 25) and MRI (1). Therefore, it is likely that the diagnostic modalities used in the present study may have underestimated the real prevalence of CVMs in our population.

The vertebral column outgrows the spinal cord in length, and the degree of elongated growth of spinal cord segments varies regionally (26). Thus, positions of most spinal cord segments reside in the vertebral canal cranial to the vertebra of the same number. This phenomenon is most pronounced in the caudal lumbar and sacrocaudal segments of the spinal cord. Generally, the three sacral spinal cord segments lie within the fifth lumbar vertebral foramen, albeit with some variation between breeds: in small breeds, these segments extend approximately one vertebra further caudally (2, 26). As a result, the segments involved in the withdrawal reflex of the pelvic limbs (L6–S2) likely reside in the vertebral canal caudal to the L4 vertebra. Therefore, the withdrawal reflex could be decreased/eliminated by an IVDE at the L4–L5 intervertebral space or caudally. For this reason, in order to compare clinical findings (normal, decreased, or absent withdrawal reflexes) with the presence/absence of LS changes, the L4 vertebrae were taken as a cut-off point to classify the population into 2 groups: 61.3% of dogs were diagnosed with an IVDE cranial to L4 (group A) and 38.8% with an IVDE caudal to L4 (group B).

Forty-one out of the 73 FBs with LS IVDP (30 in group A and 11 in group B) had a bilaterally normal withdrawal reflex and none of them showed LS pain upon neurological examination. Therefore, in all these dogs, LS IVDP appears to be asymptomatic. This finding is in accordance with the 52.8% of neurologically normal FBs with LS IVDH reported in the literature (13).

Thirty-two out of 73 dogs with LS IVDP (13 in group A and 19 in group B) had a decreased or absent withdrawal reflex. In group A, 2 dogs were paraplegic (one graded 4 and the other graded 5); and the remaining 11 dogs showed variable degrees of paraparesis. Paraplegic dogs have increased odds of spinal shock, a condition characterized by impaired spinal reflexes and muscle tone caudal to an injury to the spinal cord, compared to dogs with persistent motor function (2, 27). Therefore, the impaired withdrawal reflex in the two paraplegic dogs was likely a consequence of the spinal shock caused by IVDE rather than due to the LS IVDP. In contrast, the 11 paraparetic dogs are more likely to have shown a decreased or absent withdrawal reflex as a consequence of LS IVDP. This is an important fact from a clinical point of view.

When performing a neurological examination in FBs, clinicians should be aware that chronic neurological deficits caused by LS IVDP might interfere with the neurological deficits due to acute IVDE, potentially leading to a wrong localization. In group B, a decreased or absent withdrawal reflex could be due to both acute IVDE and chronic LS IVDP. Both types of IVDH could contribute to a clinical presentation. This fact might not have an important impact in making immediate surgical decisions, since IVDE is likely to play a major role. However, a possible persistent flexor impairment in the follow-up could be erroneously attributed to irreversible spinal cord damage at the level of IVDE rather than to IVDP—especially in dogs where an L7 nerve root involvement has been seen using MRI.

LS pain is reported to be the most frequent and first presenting clinical sign in dogs diagnosed with LS degenerative stenosis (8). Approximately half of the FBs with LS IVDP in our population were graded at least 2 regarding the severity of IVDP, but only 5.5% of the FBs with LS IVDP in our population exhibited LS spinal pain upon neurological examination. One possible explanation could be that most dogs presented with severe thoracic or lumbar hyperesthesia related to acute IVDE and the pain possibly arising at the LS junction could be masked or overlooked under neurological examination. Therefore, it is advisable to carefully evaluate FBs with thoracic or lumbar IVDE for the presence of LS hyperesthesia.

The main limitations of this study are due to its retrospective nature. The first concerns the different MRI protocols used to investigate the LS region. This lack of standardization may have influenced the assessment of LS changes, especially with regard to nerve root involvement. However, as an inclusion criterion, all dogs were at least examined in the sagittal plane, which is considered an accurate approach in evaluating canalar and foraminal involvement. Another limitation is the lack of clinical follow-up. A long-term assessment of the hind limb withdrawal reflex after recovery from IVDE might have allowed for gathering of additional information about the role of LS IVDP in FBs with an IVDE caudal to L4.

In conclusion, LS IVDP is frequently reported in FBs, with or without cranial intervertebral foraminal stenosis, nerve root involvement, or CVMs, which often causes neither deficits upon neurological examination nor evident LS pain. In some dogs, chronic LS IVDP may cause neurological deficits that may lead to incorrect neurolocalization, representing a possible confounding factor for clinicians even in young and middle-aged subjects. Therefore, FBs need to be carefully examined at the level of the LS junction even when a patient is presenting with thoracic or lumbar IVDE.

## Data availability statement

The original contributions presented in the study are included in the article/Supplementary material, further inquiries can be directed to the corresponding author.

## Ethics statement

Ethical approval was not required for the studies involving animals in accordance with the local legislation and institutional requirements because the animal study was a retrospective study. Written informed consent was not obtained from the owners for the participation of their animals in this study because it was a retrospective study on routine clinical work-up.

## Author contributions

CLR: Data curation, Formal analysis, Investigation, Resources, Writing – original draft, Writing – review & editing. SM: Formal analysis, Writing – review & editing. AC: Formal analysis, Writing – review & editing. TD: Formal analysis, Resources, Writing – review & editing. CR: Data curation, Investigation, Writing – review & editing. SS: Writing – review & editing. MB: Conceptualization, Formal analysis, Project administration, Resources, Supervision, Writing – original draft, Writing – review & editing.

## Footnotes

^1^ www.horosproject.com

## References

[1] Mai W. Diagnostic MRI in dogs and cats. Boca Raton: CRC Press (2018). 766 p.

[2] De Lahunta A Glass E Kent M. Veterinary neuroanatomy and clinical neurology. 5th ed. Philadelphia: Elsevier (2021).

[3] Da Costa RC De Decker S Lewis MJ Volk H. Canine spinal cord injury consortium (CANSORT-SCI). Diagnostic imaging in intervertebral disc disease. Front Vet Sci. (2020) 7:588338. doi: 10.3389/fvets.2020.588338, PMID: 33195623 PMC7642913

[4] Brisson BA. Intervertebral disc disease in dogs. Vet Clin North Am Small Anim Pract. (2010) 40:829–58. doi: 10.1016/j.cvsm.2010.06.001 20732594

[5] Fenn J Olby NJ. Canine spinal cord injury consortium (CANSORT-SCI). Classification of intervertebral disc disease. Front Vet Sci. (2020) 7:579025. doi: 10.3389/fvets.2020.579025, PMID: 33134360 PMC7572860

[6] Mayousse V Desquilbet L Jeandel A Blot S. Prevalence of neurological disorders in French bulldog: a retrospective study of 343 cases (2002-2016). BMC Vet Res. (2017) 13:212. doi: 10.1186/s12917-017-1132-2, PMID: 28676057 PMC5497356

[7] Smolders LA Bergknut N Grinwis GC Hagman R Lagerstedt AS Hazewinkel HA. Intervertebral disc degeneration in the dog. Part 2: chondrodystrophic and non-chondrodystrophic breeds. Vet J. (2013) 195:292–9. doi: 10.1016/j.tvjl.2012.10.011, PMID: 23154070

[8] Worth A Meij B Jeffery N. Canine degenerative lumbosacral stenosis: prevalence, impact and management strategies. Vet Med. (2019) 10:169–83. doi: 10.2147/VMRR.S180448, PMID: 31819860 PMC6875490

[9] Kranenburg HJ Grinwis GC Bergknut N Gahrmann N Voorhout G Hazewinkel HA. Intervertebral disc disease in dogs – Part 2: comparison of clinical, magnetic resonance imaging, and histological findings in 74 surgically treated dogs. Vet J. (2013) 195:164–71. doi: 10.1016/j.tvjl.2012.06.001, PMID: 22795604

[10] Suwankong N Voorhout G Hazewinkel HA Meij BP. Agreement between computed tomography, magnetic resonance imaging, and surgical findings in dogs with degenerative lumbosacral stenosis. J Am Vet Med Assoc. (2006) 229:1924–9. doi: 10.2460/javma.229.12.1924, PMID: 17173530

[11] Dewey CW da Costa RC. Canine and Feline Neurology. 3rd edn. Ames: John Wiley & Sons (2016).

[12] De Risio L Thomas WB Sharp NJ. Degenerative lumbosacral stenosis. Vet Clin North Am Small Anim Pract. (2000) 30:111–32, vi. doi: 10.1016/s0195-5616(00)50005-9 10680211

[13] Bertram S Ter Haar G De Decker S. Congenital malformations of the lumbosacral vertebral column are common in neurologically normal French bulldogs, English bulldogs, and pugs, with breed-specific differences. Vet Radiol Ultrasound. (2019) 60:400–8. doi: 10.1111/vru.12753, PMID: 31050057

[14] Lecourtois C Baudin-Tréhiou C Blond L. Lumbosacral endplate contour defect is frequently observed concurrent with other lumbosacral abnormalities on spinal CT of French bulldogs. Vet Radiol Ultrasound. (2023) 64:813–22. doi: 10.1111/vru.13271, PMID: 37366604

[15] Balducci F Canal S Contiero B Bernardini M. Prevalence and risk factors for presumptive ascending/descending myelomalacia in dogs after thoracolumbar intervertebral disk herniation. J Vet Intern Med. (2017) 31:498–504. doi: 10.1111/jvim.14656, PMID: 28144987 PMC5354033

[16] Lampe R Foss KD Hague DW Oliveira CR Smith R. Dynamic MRI is reliable for evaluation of the lumbosacral spine in healthy dogs. Vet Radiol Ultrasound. (2020) 61:555–65. doi: 10.1111/vru.12891, PMID: 32574428

[17] Jones JC Davies SE Werre SR Shackelford KL. Effects of body position and clinical signs on L7-S1 intervertebral foraminal area and lumbosacral angle in dogs with lumbosacral disease as measured via computed tomography. Am J Vet Res. (2008) 69:1446–54. doi: 10.2460/ajvr.69.11.1446, PMID: 18980426

[18] Schwarz T Saunders J. Veterinary computed tomography. 1st ed. Chichester: John Wiley & Sons Ltd (2011).

[19] Robertson I Thrall DE. Imaging dogs with suspected disc herniation: pros and cons of myelography, computed tomography, and magnetic resonance. Vet Radiol Ultrasound. (2011) 52:S81–4. doi: 10.1111/j.1740-8261.2010.01788.x, PMID: 21392160

[20] Flückiger MA Damur-Djuric N Hässig M Morgan JP Steffen F. A lumbosacral transitional vertebra in the dog predisposes to cauda equina syndrome. Vet Radiol Ultrasound. (2006) 47:39–44. doi: 10.1111/j.1740-8261.2005.00103.x, PMID: 16429983

[21] Lackmann F Forterre F Brunnberg L Loderstedt S. Epidemiological study of congenital malformations of the vertebral column in French bulldogs, English bulldogs and pugs. Vet Rec. (2022) 190:e509. doi: 10.1002/vetr.509, PMID: 34021609

[22] Gong H Slunsky P Klass LG Brunnberg L. Prevalence of lumbosacral transitional vertebrae in dogs in Berlin. Pol J Vet Sci. (2020) 23:261–5. doi: 10.24425/pjvs.2020.133641, PMID: 32627986

[23] Kuricová M Ledecký V Kvetková J Lipták T. Vertebral malformations in French bulldogs. J Fac Vet Med Istanbul Univ. (2017) 43:1–153. doi: 10.16988/iuvfd.322981

[24] Brocal J De Decker S José-López R Guevar J Ortega M Parkin T. Evaluation of radiography as a screening method for detection and characterisation of congenital vertebral malformations in dogs. Vet Rec. (2018) 182:573. doi: 10.1136/vr.104388, PMID: 29519855

[25] Corlat L Blanco B Lucena R Pj G Mirò F Novales M. Congenital vertebral malformations in French bulldogs: X-ray vs computed tomography. Bull UASVM Vet Med. (2017) 74:11508. doi: 10.15835/buasvmcn-vm

[26] Evans HE De Lahunta A. Miller’s anatomy of the dog. 4th ed. St. Louis: Elsevier (2013).

[27] McBride R Parker E Garabed RB Olby NJ Tipold A Stein VM. Developing a predictive model for spinal shock in dogs with spinal cord injury. J Vet Intern Med. (2022) 36:663–71. doi: 10.1111/jvim.16352, PMID: 35001437 PMC8965241

