# Supplementary material for: Prevalence, MRI findings, and clinical features of lumbosacral intervertebral disc protrusion in French Bulldogs diagnosed with acute thoracic or lumbar intervertebral disc extrusion
Source: Front Vet Sci. 2023 Nov 23;10:1302418. doi: 10.3389/fvets.2023.1302418 (PMC10702215; doi:10.3389/fvets.2023.1302418)
Supplement: Supplementary file 1 [file Table_1.DOCX]

Supplementary Material

Prevalence, MRI findings, and clinical features of lumbosacral intervertebral disc protrusion in French Bulldogs diagnosed with acute thoracic or lumbar intervertebral disc extrusion.

Claudia La Rosa^1*^, Simona Morabito^1,2^, Andrea Carloni^1,2^, Tommaso Davini^1^, Carlotta Remelli^1^, Swan Specchi^1,2^, Marco Bernardini^1,3^

^1^Anicura I Portoni Rossi Veterinary Hospital, Zola Predosa, Bologna, Italy

^2^Antech Imaging Service, Fountain Valley, California, USA

^3^Department of Animal Medicine, Productions and Health, University of Padua, Legnaro, Italy

*** Corresponding Author**Claudia La Rosa
mail to: claudia.la.rosa@anicura.it

# Supplementary Table 1. MRI sequences available in all dogs included.

| Plane  Sequences | Sagittal | Dorsal | Transverse |
| --- | --- | --- | --- |
| FSE-T2W | 80 (100%) | 17 (21.3%) | 30 (37.5%) |
| FSE-T1W | 14 (17.5%) | 4 (5%) | 18 (22.5%) |
| STIR | 0 (0%) | 69 (86.3%) | 1(1.3%) |
| FLAIR-T2W | 0 (0%) | 1 (1.3%) | 1 (1.3%) |
| PD | 1 (1.3%) | 0 (0%) | 2 (2.5%) |
| GRE-T2* | 0 (0%) | 3 (3.8%) | 0 (0%) |
| isometric 3DT1MPRAGE | 7 (8.8%) | | |

FSE, Fast spin echo; STIR, Short-tau Inversion Recovery; FLAIR, Fluid-attenuated Inversion Recovery; PD, Proton density; GRE, Gradient-echo; 3DT1MPRAGE, 3D pre- and postcontrast Magnetization Prepared Rapid Gradient Echo.
